# Patients’ experiences with multidose drug dispensing: a cross sectional study

**DOI:** 10.1007/s11096-018-0749-y

**Published:** 2018-11-26

**Authors:** Bram J. Mertens, Henk-Frans Kwint, Rob J. van Marum, Marcel L. Bouvy

**Affiliations:** 1 Division of Pharmacoepidemiology and Clinical Pharmacology, Utrecht Institute for Pharmaceutical Sciences, University Utrecht, Utrecht, The Netherlands; 2 SIR Institute for Pharmacy Practice and Policy, Theda Mansholtstraat 5b, 2331 JE Leiden, The Netherlands; 3 Geriatric Department, Jeroen Bosch Hospital, ‘s-Hertogenbosch, The Netherlands; 4 Department of General Practice and Elderly Care Medicine, EMGO Institute for Health and Care Research, VU University Medical Center, Amsterdam, The Netherlands

**Keywords:** Adherence, Community pharmacy, Dosing aids, Multidose drug dispensing, Patients’ experiences, The Netherlands

## Abstract

*Background* Automated multidose drug dispensing is used to support patients with their medication management. Though multidose drug dispensing systems are frequently used, little is known about patients’ experiences with multidose drug dispensing systems. *Objective* To explore patients’ experiences with the initiation and use of multidose drug dispensing systems. *Setting* A survey was carried out with patients using multidose drug dispensing systems through three community pharmacies. *Method* A semi-structured interview protocol was designed based on existing literature and a pilot study. *Main outcome measures* The main outcome measures were (1) patients’ experiences with initiating multidose drug dispensing systems and (2) patients’ experienced advantages and disadvantages of multidose drug dispensing systems. *Results* The start of multidose drug dispensing was discussed with 76% of the patients (n = 62). Ninety percent of patients expressed the opinion that the multidose drug dispensing system supported them with their medication management. Sixty patients reported 110 advantages, which can be organized into the following categories: improved medication adherence and medication safety (59%); patient’s convenience (40%); and other (1%). Sixty-nine percent of patients reported no disadvantages, 24% had problems opening the bags or outer packaging and 13% had problems with the legibility of the printed text on the bag. *Conclusion* In concordance with the Dutch guideline, patients are generally involved in the decision to initiate an multidose drug dispensing system. Patients are very satisfied using the system and report multiple advantages. Multidose drug dispensing systems may be further improved by simplifying the manual opening of the bags and improving the legibility of the text on the bags.

## Impacts on practice

- The initiation of multidose drug dispensing systems improves patients’ reported medication management.
- The majority of Dutch patients is very satisfied with the use of an multidose drug dispensing.
- The ease of opening of individual plastic bags and the legibility of the printed text on multidose drug dispensing systems in the Netherlands should be improved.

## Introduction

Despite the extensive research and attention of healthcare providers, patients’ non-adherence to prescribed medication regimens remains a significant issue in healthcare [1]. Non-adherence can be divided in intentional and unintentional non-adherence [2]. Intentional non-adherent patients deliberately decide not to adhere to their drug therapy. Interventions that target intentional non-adherence focus on the patient’s perceptions on medication [2]. Unintentional non-adherent patients are willing but unconsciously fail to adhere to their drug therapy. The capacity to adhere to the medication regimen may be diminished by a wide variety of reasons, e.g., a decline in cognitive function, polypharmacy, a change in appearance of outer packaging, or impaired manual dexterity [3–5]. Especially for unintentional non-adherence, the use of dosing aids such as multi-compartment compliance aids (MCAs) or automated multidose drug dispensing (MDD) systems can be an option [6].

In the Netherlands, MDD systems are the preferred type of dosing aid, and the number of MDD users is rapidly increasing [7]. In the Netherlands, approximately 12% of patients aged ≥ 65 years use an MDD system [8, 9]. In MDD systems, all solid medication intended for one dosing moment (e.g. capsules and tablets) is robot-packed in plastic disposable bags. Patient identification, content and designated time of intake is printed on the bags [10, 11]. MDD systems are generally dispensed for a period of one to 3 weeks.

The Dutch guideline for patients using multi-compartment dosing aids recommends that patients who have difficulty adhering to their dosing regimens be primarily supported by simplification of complex dosing regimens, automatic refills of chronic medication or written dosing schemes [7]. If these options fail, an MDD system can be initiated. Shared decision making during this process is emphasised.

Little is known about patients’ experiences and satisfaction with dosing aid. A small qualitative study of 15 users of various forms of dosing aids in the United Kingdom revealed that patients have divergent opinion [12]. Some experienced the use of a dosing aid as a supportive tool that helped them remain independent, while others perceived a loss of independence. Remarkably, patients stated that the initiation of the dosing aid was rarely discussed with them. These divergent results were supported by a second qualitative study among patients who lived in very sheltered housing in Scotland [5]. Patients’ opinions differed regarding shared decision making, independence, medication knowledge and their confidence in managing their medication. Corresponding results were found in a survey among users of MCA admitted to a medical ward [13]. However, contrasting results were found in a questionnaire study that focused on patients’ experiences with MDD systems [14]. Most patients were very satisfied using an MDD system. Though MDD systems are used extensively in the Netherlands, these patients’ experiences with MDD systems are not well understood.

## Aim of the study

The aim of this study was to explore patients’ experiences with the initiation and use of MDD systems.

## Ethics approval

In accordance with the Dutch Medical Research Involving Human Subjects Act, no formal ethical approval was needed to conduct this study. Patients gave written informed consent before the interview was conducted. In order to protect the patients’ privacy, only age and gender were documented.

## Method

### Study design

This was a cross-sectional study between February and April 2016 among MDD users from three community pharmacies in the Netherlands. Patients were interviewed using a structured interview protocol (see “Appendix”). The interview focused on the patient’s experiences with MDD systems. The initial version of the interview protocol (drafted by BM and SB) contained all questions from earlier studies which explored the patients’ experiences and attitudes of MCAs or MDD systems [12–14]. Next, duplicate questions and questions not related to the use of the MDD systems were deleted. Closed-ended questions about specific advantages or disadvantages of MDD systems were replaced by open-ended questions on the advantages or disadvantages of an MDD system. Subsequently, the interview protocol was supplemented with questions derived from the recommendations from the Dutch guideline for patients using multi-compartment dosing aids [7]. HFK and MB commented on the drafted interview protocol. Based on the comments, the protocol was adjusted and tested in a small pilot among five patients. After the pilot, the protocol was once more adjusted. Ambiguous questions were altered and clarified. The final interview protocol contained both open-ended and closed questions.

### Patients

The patients’ community pharmacist first screened all users of MDD systems on inclusion and exclusion criteria. Patients over 18 years of age who received their drugs via an MDD system were eligible for inclusion. Patients with known severe cognitive impairment, who received palliative care or home care responsible for the administration of their medication, were excluded from participation. An independent researcher (SB) was temporarily seconded in the community pharmacy and invited patients to participate by telephone from their community pharmacy. If interested in study participation, patients received written study information. For the interview, patients were visited at home and before the start of the interview written informed consent was obtained. During the interview answers were written down by the independent researcher (SB). No audio recording was performed. Patients did not receive any payment or incentive for their participation and individual results were not shared with employees of the community pharmacy.

### Sample size

The study was conducted in three average sized community pharmacies. We estimated that a total number of 405 patients would be eligible for inclusion. For a representative sample with an alfa of 10% and a power of 90%, 59 patients were needed.

### Data coding and analysis

Answers from the five patients in the pilot phase were excluded from the analysis. Answers to open-ended questions were coded and assigned by two researchers (SB and BM). Discrepancies in coding were addressed in a consensus meeting. All data were analysed using statistical software (SPSS version 23.0; SPSS Inc., Chicago, IL, USA). Descriptive statistics were used for basic characteristics. Normally distributed data are presented with means and standard deviations (SD). For non-normally distributed data, median scores with the interquartile range (IQR) are presented. Answers to all questions are presented with absolute numbers and percentages.

## Results

One hundred forty-seven patients were invited to participate, of which 62 (42%) gave informed consent. Patient characteristics are shown in Table 1. Median age was 79.5 and 57% (n = 35) was female. The mean time per interview was 20 min (SD = 8). Patients indicated that pharmacists most often initiated the MDD system (22.6%, n = 14), followed by the GP (14.5%, n = 9) and the patient’s family (14.5%, n = 9). Home care (12.9%, n = 8), the patient (6.5%, n = 4) and a medical specialist (4.8%, n = 3) also initiated MDD systems. 24.2% (n = 15) of the patients could not remember who had initiated their MDD systems. The start of an MDD system was discussed with 76% (n = 47) of the patients. MDD systems were initiated because the patient had a decreased medication management capacity (50% n = 31), patient convenience (19%, n = 12), forgetfulness (15%, n = 9), practical problems related to the opening of the inner or outer packaging of medication (3%, n = 2) and to anticipate on further decline of the patient’s health (3%, n = 2). Ten percent (n = 6) of the patients did not remember the reason for the initiation of the MDD system. Ninety percent (n = 56) of patients were of the opinion that the MDD system supported them with their medication use, and 76% (n = 47) indicated that the MDD system had improved their medication management. The median satisfaction rate for the use of an MDD system was 8 (IQR; 8–9) on a scale of 1–10 (1 extremely unsatisfied and 10 extremely satisfied). Two patients rated the use of an MDD system a 5, and none of the patients preferred to return to manual dispensing.

**Table 1:** Patient characteristics of the included patients

Patient characteristics (n = 62)	
Female; *n* (%)	35 (57%)
Age; median (IQR)	79.5 (74–85)
Years MDD system in use; median (IQR)	1.7 (0.5–4.1)
Number of drugs dispensed via MDD system; mean (SD)	7.8 (2.4)
Number of manually dispensed drugs; median (IQR)	4 (1–5)

*n* number, *IQR* interquartile range, *MDD* multidose drug dispensing, *SD* standard deviation

In total, 60 (97%) patients reported 110 advantages, and 2 patients (3%) reported no advantages. Most advantages were classified under the domain medication safety and adherence (59%, n = 65) (see Table 2). Thirty-seven disadvantages were reported by 19 (31%) patients, and 43 patients (69%) reported no disadvantages. Most frequently reported disadvantages concerned difficulties with the opening of MDD systems (41%, n = 15).

**Table 2:** The reported advantages addressed to three domains and reported disadvantages

Domain	Advantages	n = 110 (100%)
Medication safety and adherence	I don’t make mistakes with my medication anymore	44 (40%)
	It helps me not to forget to take my medication	15 (14%)
	I have a better overview over my medication	6 (5%)
Patient’s convenience	I don’t have to sort my medication anymore	15 (14%)
	The medication can easily be taken on an excursion or holiday	12 (11%)
	The medication is automatically prescribed and dispensed	10 (9%)
	The medication is periodically delivered at home	3 (3%)
	I spent less time taking my medication	2 (2%)
	I need less space to store my medication	1 (1%)
	The bags are more easy to open compared to blisters	1 (1%)
Other	There is less waste from packaging	1 (1%)

	Disadvantages	37 (100%)
	I have problems opening the outer packaging or pouches	15 (41%)
	I can’t read the printed text on the pouches	8 (22%)
	It’s difficult to identify the medication	5 (14%)
	It’s difficult that not all medication is dispensed via the MDD system	3 (8%)
	Medication is only dispensed for a short period of time	2 (5%)
	The home delivery of the medication can be a problem	1 (3%)
	There is more waste from packaging	1 (3%)
	The MDD system contains generic medication	1 (3%)
	The MDD system contained medication that shouldn’t be in the MDD system	1 (3%)

## Discussion

For most patients, MDD systems were initiated after shared decision making. Patients were very satisfied with the use of MDD systems and expressed the belief that the system supported and improved their ability to use medication appropriately. However, disadvantages were also reported. These included difficulties opening the packaging and legibility of the printed text on the bags.

The divergent results found in the studies conducted in the United Kingdom and Scotland were not replicated in the present study [5, 12, 13]. In our study, patients were generally involved in the decision to initiate an MDD system and patient satisfaction with MDD systems was found to be high. Our results were more consistent with the results found by Bardage et al. [14] among users of MDD systems in Sweden. A direct comparison with Nunney et al. and Stewart et al. is difficult as, due to the qualitative study design, absolute numbers and percentages are lacking. In addition, Nunney et al. and Stewart et al. included residents who lived in (very) sheltered housing in contrast to our study where patients lived in the community without home care. However, in accordance with the both studies, the importance of the recommendation only to initiate an MDD system after a shared decision became again apparent in our study. The two patients who rated the use of an MDD system with a 5 were not consulted before the initiation. This emphasises the importance of involving the patient in the decision to initiate an MDD system.

Besides the improved medication adherence, the use of MDD systems has also some drawbacks. The patient’s medication knowledge seems to diminish, not all the patient’s medication can be dispensed via MDD systems and dispensing medication via an MDD system is more costly compared to manual dispensing. In addition, there are concerns about the drug stability of medication outside its original packaging [15]. If other interventions fail or are not appropriate (e.g. simplification of the drug medication regimen, the use of drug reminder charts, or the use of reminder alarms), an MDD system can be initiated. In half of the patients, an MDD system was initiated because the patient had a decreased medication management capacity. Patients with a decreased medication management capacity are willing to adhere but unconsciously fail because of a wide variety of reasons [3]. For these patients the initiation of an MDD system can be seen as appropriate.

However, also one quarter of the patients indicated that the initiation of an MDD system did not improve their medication management. This raises the question of why these patients were using an MDD system. When initiating dosing aids, it’s advised to perform an individualized suitability assessment before the initiation of dosing aid [7, 15, 16]. Ideally, an intervention is used in which the patient continues to receive the medication via original packaging to maintain the patient’s self-efficacy [7, 15, 16]. Our findings were in line with a different study among patients who were about to start with an MDD system in which 30% of the patients did not have a decreased medication management capacity [3]. No improvement can be expected among these patients. In the Netherlands, the use of MDD systems is reimbursed by health insurance and is about five times more expensive than manual dispensing. Initiating an MDD system solely for convenience is, therefore, inappropriate. For instance, one frequently cited advantage of MDDs—that medication can be easily taken on an excursion—can also be conferred with the use of a small pillbox. Therefore, other possible solutions must be explored before an MDD system is initiated.

Similar disadvantages to those found in previous studies were reported. Approximately 25% of the MDD users experienced difficulties with opening of the MDD system, though innovations like die-punched tear-lines have been introduced in the past years. These practical problems are not specific to MDD users, as can be concluded from the fact that similar percentages are also found among older adults who use original packaging [4, 17]. However, the introduction of an MDD system has been described as a strategy to overcome these practical problems [12, 14]. Whether the introduction of an MDD system solves these issues in reality remains unclear, as multiple patients continued to experience difficulties opening the MDD system. Especially patients with a reduced manual dexterity or reduced vision are prone to encounter problems regarding the packaging of their medication. On the other hand, there was one patient who described the opening of the MDD system as an advantage. It can be concluded that the MDD system’s success as a solution depends on the individual patient’s situation. When the decision is made to start an MDD system, patients’ experiences must also be periodically evaluated, as their health status is expected to decline over time [3]. In general, the user friendliness of MMD systems might be improved with additional strategies which are targeted on patients with a reduced manual dexterity.

This study had several strengths. First, interviews were conducted by an independent interviewer at the patients’ homes. This interviewer was an independent research associate unconnected to the community pharmacy. In this way, the risk of eliciting merely socially desirable answers was minimized. Second, this study explored the patients’ experiences with both the initiation and continued use of MDD systems. Third, only patients who were responsible for their own medication management were included. Among these patients, MDD systems are intended to improve the patient’s medication management. However, as earlier discussed, not all patients experienced an improvement in their medication management.

This study also had some potential limitations. First, patients were recruited on a voluntary basis, which might have introduced selection bias. Patients who were satisfied with the use of an MDD system might have been more willing to participate. Consequently, those with negative experiences with MDD systems might be underrepresented. In theory, patients who use an MDD system longer are more satisfied, but no correlation between the number of years patients used an MDD system and their satisfaction was found. That said, unsatisfied patients who had already returned to manual dispensing were not included in this study. Patients rarely return to manual dispensing in practice. Second, our study was designed as a structured interview with no room for in-depth questioning. The results show that most patients are satisfied but this does not account for all patients. To explore more in-depth the experiences and attitudes of the unsatisfied patients a qualitative study should be performed. Third, patients who received professional home care for the administration of the medication were excluded from participation. This group of patients might be especially likely to encounter problems with the use of MDD systems. However, these patients are supported with their medication use by home care workers who are trained and familiar with MDD systems. At last, the study was performed in only three community pharmacies all located in an urban area. All three pharmacies indicated to adhere to the Dutch guideline. Three pharmacies is a relatively low number of participating pharmacies. Possibly, results from more different pharmacies located in rural areas or who don’t adhere to the Dutch guideline might differ.

## Conclusion

Patients are generally involved in the decision to initiate an MDD system, however, there is still room for improvement. For most patients, the use of an MDD system seems appropriate, but there are also patients in who the use of an MDD system might be questionable. Patients are very satisfied using the system and report multiple advantages. MDD systems may be further improved by simplifying the manual opening of the bags and improving the legibility of the text on the bags. In order to be able to generalize the found results for all MDD users, results must be repeated with patients from different pharmacies.
